# Effect of edaphoclimate on the resin glycoside profile of the ruderal *Ipomoea parasitica* (Convolvulaceae)

**DOI:** 10.1371/journal.pone.0305003

**Published:** 2024-08-08

**Authors:** Edmi Pérez-Sanvicente, Ismael León-Rivera, Alexandre T. Cardoso-Taketa, Irene de la C. Perea-Arango, Patricia Mussali-Galante, Susana Valencia-Díaz

**Affiliations:** 1 Centro de Investigación en Biotecnología (CEIB), Universidad Autónoma del Estado de Morelos, Cuernavaca, Morelos, México; 2 Centro de Investigaciones Químicas, IICBA, Universidad Autónoma del Estado de Morelos, Cuernavaca, Morelos, México; University of California Riverside, UNITED STATES

## Abstract

The latex of *Ipomoea* (Convolvulaceae) is a source of a special kind of acylsugars called resin glycosides, which are highly appreciated because of their biological activities (*i*.*e*. laxative, antimicrobial, cytotoxic etc.). Most research has been conducted in perennials with tuberous roots, where resin glycosides are stored. However, their content and variation are unknown in annual vines that lack this type of root, such as in the case of *Ipomoea parasitica*. This species contains research/biological and human value through its fast growth, survival in harsh environments, and employment in humans for mental/cognitive improvements. These qualities make *I*. *parasitica* an ideal system to profile resin glycosides and their variations in response to edaphoclimate. Topsoil samples (0–30 cm depth) and latex from petioles of *I*. *parasitica* were collected in two localities of central Mexico. The latex was analyzed through UHPLC-ESI-QTOF, and soil physico-chemical characteristics, the rainfall, minimum, average, and maximum temperatures were recorded. We also measured canopy (%), rockiness (%), and plant cover (%). A Principal Component Analysis was conducted to find associations between edaphoclimate and the resin glycosides. Forty-four resin glycosides were found in the latex of *I*. *parasitica*. Ten correlated significantly with three components (47.07%) and contained tetrasaccharide, pentasaccharide, and dimers of tetrasaccharide units. Five resin glycosides were considered constitutive because they were in all the plants. However, exclusive molecules to each locality were also present, which we hypothesize is in response to significant microhabitat conditions found in this study (temperature, clay content, pH, and potassium). Our results showed the presence of resin glycosides in *I*. *parasitica* latex and are the basis for experimentally testing the effect of the conditions above on these molecules. However, ecological, molecular, and biochemical factors should be considered in experiments designed to produce these complex molecules.

## Introduction

Glycolipids are part of the thylakoid membrane and maintain stability in the photosynthetic machinery. They confer thermotolerance to plants when they are exposed to high temperatures (38°C), keep membrane fluidity under phosphate deficiency [1], and play an essential role in plant interactions, acting as a defense signal molecule when leaves are damaged by biotic and abiotic stress [2]. The latex of Convolvulaceae (“morning glory” family) contains a mixture of glycolipids called resin glycosides, which are a kind of acylsugars [3–5], with linear and branched acylated chains along the sugar cores [6]. Several resin glycosides of *Ipomoea* species (Convolvulaceae) have been characterized [4] and are highly appreciated because of their biological activities (i.e., allelopathic, antidepressant, cytotoxic, insecticide) [4, 7–10]. Some species are even cultivated traditionally because the purgative activity of their resin glycosides, such as *Ipomoea purga* (Wender.) Hayne [11].

It is well known that abiotic stressors like extreme temperatures, salinity, drought, nutrient deficiency, and soil physical characteristics limit the metabolism and growth of plants [12–14]. In particular, soil characteristics, which are closely related to climatic conditions [15], influence the growth rate of roots and stems [16], leaf expansion, stomatal conductance [17], and leaf size [18].

The variation of soil characteristics like the organic matter, pH, soil particle type, P, and N, as well as drought and temperature, modify the secondary metabolite production [12, 19–21]. Concerning resin glycosides, no studies have related specific abiotic factors to changes in the content of these molecules; however, their yield and mixture in *Ipomoea tyrianthina* Lindl. [8] vary according to the geographic distribution, reflecting indirectly the environmental conditions of the habitat where the plants of this species grow. On the other hand, the resin glycosides of *I*. *batatas* periderm change yearly [22], which can be interpreted because of environmental conditions.

The resin glycosides are mainly located in the latex of underground storage organs of some *Ipomoea* perennial herbs [4], like *I*. *stans* Cav. and the vine *I*. *orizabensis* [23, 24]. However, other species closely related to those mentioned lack tuberous roots [25, 26] and have not been studied enough. Such is the case of the annual species *Ipomoea parasitica*, whose latex and its resin glycosides were mainly located in aerial structures.

*Ipomoea parasitica* is a ruderal species that grows annually in changing and stressful environments, is considered ornamental, and is used to improve human mental/cognitive skills [27]. Despite having ethnobotanical importance and being a potential source of resin glycosides, there are scarce phytochemical studies about this species [28, 29]; just one study reported some resin glycosides as a mixture of two disaccharides or mono and a trisaccharide moiety bonded to jalapinolic acid [29]. The characterization and identification of resin glycosides are challenging due to their structural complexity and high molecular weight; additionally, there is insufficient information about the environmental elicitors affecting their production. Hence, this study aims to determine the resin glycoside profile in the latex of *I*. *parasitica* and to find possible associations between this species’ resin glycoside content and edaphoclimate.

## Materials and methods

### Study area

The plant material was collected from 11 plants of *I*. *parasitica* (mean ± 1SD: 3.02±1.76 m of height) that came from two localities that belong to the Chichinautzin Biological Corridor in Morelos, Mexico: Amatlan de Quetzalcoatl, Tepoztlan (18° 58’ 44" N, 99° 02’ 11" W, 1250–2300 m asl) and Oacalco, Yautepec (18° 53’ 09" N, 99° 03’ 38" W, 1250 m a.s.l.). According to the modified Köppen climate system [30], these localities have a semi-warm, sub-humid climate, and the rainfall occurs in summer and early autumn. Tepoztlan’s mean annual temperature is 20.7°C, and rainfall varies from 1,000 to 1,200 mm. In Yautepec, the yearly average temperature and rainfall are 22.7°C and 945.7 mm. Tropical dry forest is one of the representative vegetation types in these localities [31]. In both areas, *Ipomoea parasitica* latex samples were collected in open spaces used for cattle ranching and the cultivation of maize. Because *I*. *parasitica* grows in perturbed areas or as a weed, Amatlan de Quetzalcoatl and Oacalco communities allowed us to collect soil and plant material. One specimen of *I*. *parasitica* was deposited in the HUMO Herbarium of the Universidad Autonoma del Estado de Morelos (UAEM, voucher number 3956).

### Latex sampling and analysis

In November 2019, latex samples of 11 flowering plants of *I*. *parasitica* (n = 7, n = 4 for Tepoztlan and Yautepec, respectively) were obtained by cutting their petioles at 1 cm from the leaf base. The collected latex samples were stored in Eppendorf tubes (1 mL), placed in a portable cooler, and transported to the Centro de Investigaciones Quimicas, where they were processed. Because the samples were not frozen, we followed the collection procedure reported in other studies [32], where latex samples were dried at air temperature (20.3°C). Once dried, the latex was dissolved in a 15:85mixture of H_2_O / CH_3_OH at a final 5mg/mL concentration for chromatographic analysis. Samples were analyzed by liquid chromatography (Agilent Infinity II 1290 UHPLC) using a Zorbax Eclipse Plus C_18_ column (20°C, 2.1 x 50 mm, L.N B15169, S.N. USDAZ07084, Agilent), using as mobile phase, a gradient of H_2_O /CH_3_CN (J.T.Baker®) MS grade, both acidified with 0.5% formic acid (J.T.Baker®) and eluted in gradient mode; 0–2 min (20:80), 2–6 min (0:100), 6–8 min (0–100), 8–9 min (20:80); flow 0.25 mL/min. Detection was performed by mass spectrometry using a Q-TOF (Agilent 6545) analyzer using an ESI source and positive scan mode. The parameters were scanning range 100–3000 *m/z*, injection volume 1 μL, VCap 3500, and fragmentor 140 V, scan rate 1 spectra/sec, gas temp 300°C, gas flow 10 L/min, nebulizer 35 psig, sheath gas temp 350 L/min, sheath gas flow 11 L/min.

The data was processed by molecular characteristics for small organic molecules (MassHunter Workstation Software, Agilent, Santa Clara, CA, USA); it was considered species allowed H^+^, Na^+^, K^+^, NH_4_^+^, and neutral loss of H_2_O, maximum charge of two, an isotopic mass tolerance of 7 ppm, relative height 2.5% and a minimum number of counts 5,000. In addition to the fragmentation patterns, mass spectrometry was used to identify chemical structures, considering the *m/z* ratio. A chromatographic fraction of *Ipomoea tyrianthina* containing Tyriantin I-VII was used as a reference sample [8]. We used this species as a reference because it is near phylogenetically to *I*. *parasitica* (Series: Tyrianthinae (House) D.F. Austin) [25].

To characterize resin glycosides in the latex of *Ipomoea parasitica*, we used the mining data of glycolipids reported in the literature on mass spectra. The core of this strategy is setting the mass fragmentation of glycolipids to obtain the mass spectrum data set for target compounds, as has been reported by diverse authors [8, 33, 34]. A fingerprint analysis was performed to know the variation of resin glycosides in response to edaphoclimate factors; this analysis consists of a general screening of the metabolome of organisms instead of identifying the metabolites [35, 36].

### Soil sampling and analysis

A soil sample was collected around each plant of *I*. *parasitica*. Seven samples were taken from the topsoil (0–30 cm depth) of the Tepoztlan locality and four from Yautepec; each soil sample was the pooled small samples taken at four points around each plant. Soil samples were bagged and transported to Laboratorio de Botanica Estructural, Centro de Investigacion en Biotecnologia (CEIB), UAEM, for their analyses. Soil samples were dried at room temperature (mean ± 1SD; 16.73°C ± 5.61°C, 48h) and sieved through a mesh of stainless steel (FIICSA, No. 18, 2.00 mm). The soil parameters evaluated were: a) moisture % (gravimetric method AS-05), employing a forced convection oven Binder (FD-115) and an analytical balance (OHAUS, Pioneer PX), b) size of soil particles, and c) texture (% sand, % silt, % clay) (AS-28 and AS-09 methods respectively), employing an standard ASTM No 152H hydrometer (ROBSAN BOYOUCOS) [37], d) bulk density (BD) (AS-03 method), using a forced convection oven Binder (FD-115), a pycnometer, and analytical balance (OHAUS, Pioneer PX) [38], e) Percentages of total organic carbon (TOC) and organic carbon (OC) (AS-07 Walkley & Black method), employing a burette and an analytical balance (OHAUS, Pioneer PX), f) available phosphorus (P) (AS-10 method, Bray & Kurtz method) [39] employing a Thermo Fisher Scientific spectrophotometer (G10S UV-Vis), g) calcium (Ca^2+^) and magnesium (Mg^2+^) (AS-12 method UV-Vis Milton Roy 20D spectrophotometer), h) sodium (Na^+^) and potassium (K^+^) (AS-12 method, Corning Flame 410 flamometry), i) nitrogen % (N) (AS-08 Micro-Kjeldahl method), j) pH (AS-02 method) employing a potentiometer (Thermo scientific Orion star A215) and k) organic matter % (OM) (AS-07 method), employing a burette and an analytical balance (OHAUS, Pioneer PX) [40]. The content of Ca^2+^, K^+^, Na^+^, N, TOC %, and Mg^2+^ was determined at the Laboratorio de Suelos del Centro de Investigación y Estudios Avanzados en Cultivo de Plantas (UAEMex) following NOM-021-RECNAT-2000 [40] and at Laboratorio de Investigaciones Ambientales (CEIB, UAEM). The soil depth and rockiness of each sampled site were measured *in situ*. Soil depth was estimated with a graduated rod. While the rockiness (%) and plant cover (%) were assessed visually by placing on the soil surface a wooden square (50 × 50 cm) gridded each 25 cm. Additionally, the percentage of canopy cover (%) was estimated with a concave densitometer (Model-C, Forest Densitometers) by averaging the canopy closure of the four cardinal points (N, S, E, W).

### Climatic data

The climatic data (minimum, maximum, mean temperature, and rainfall) for Tepoztlan and Yautepec were obtained from the CONAGUA meteorological stations 17049 and 17024, respectively (smn.conagua.gob.mx). The data correspond from August to November 2019, the period in which the lifespan of *I*. *parasitica* occurs.

### Statistical analysis

Before the performance of the principal component analysis (PCA) [41], a correlation analysis [42] was carried out to eliminate redundant edaphic and climatic variables (r ≥ |0.8|). As a result, organic carbon (%), organic matter (%), and Ca^2+^ were removed, as well as the glycolipid *W* (*m/z* = 1275.7170), because of its lack of variability. The variables considered for PCA were soil moisture (%), total N (%), K^+^(ppm), Mg^2+^(ppm), Na^+^ (ppm), BD (g/cm^3^), TOC (%), silt (%), clay (%), sand (%), pH, P (ppm), soil depth (cm), rockiness (%), plant cover and canopy (%). All the climatic variables mentioned were considered for the PCA. According to their retention time and *m/z*, each resin glycoside was identified with a letter (from *A* to *AR*). PCA was based on the correlation matrix of both climatic variables and resin glycosides; the significant variables related to each PC were those with r ≥ |0.65|. Analyses were carried out in r version 4.2.1 [43], with the libraries ade4 (PCA) [44], scatterplot3d (graphics) [45]. Mann-Whitney U [42] test determined differences between the environmental variables between sites.

## Results

### Phytochemical analysis of latex

**Fig 1:**
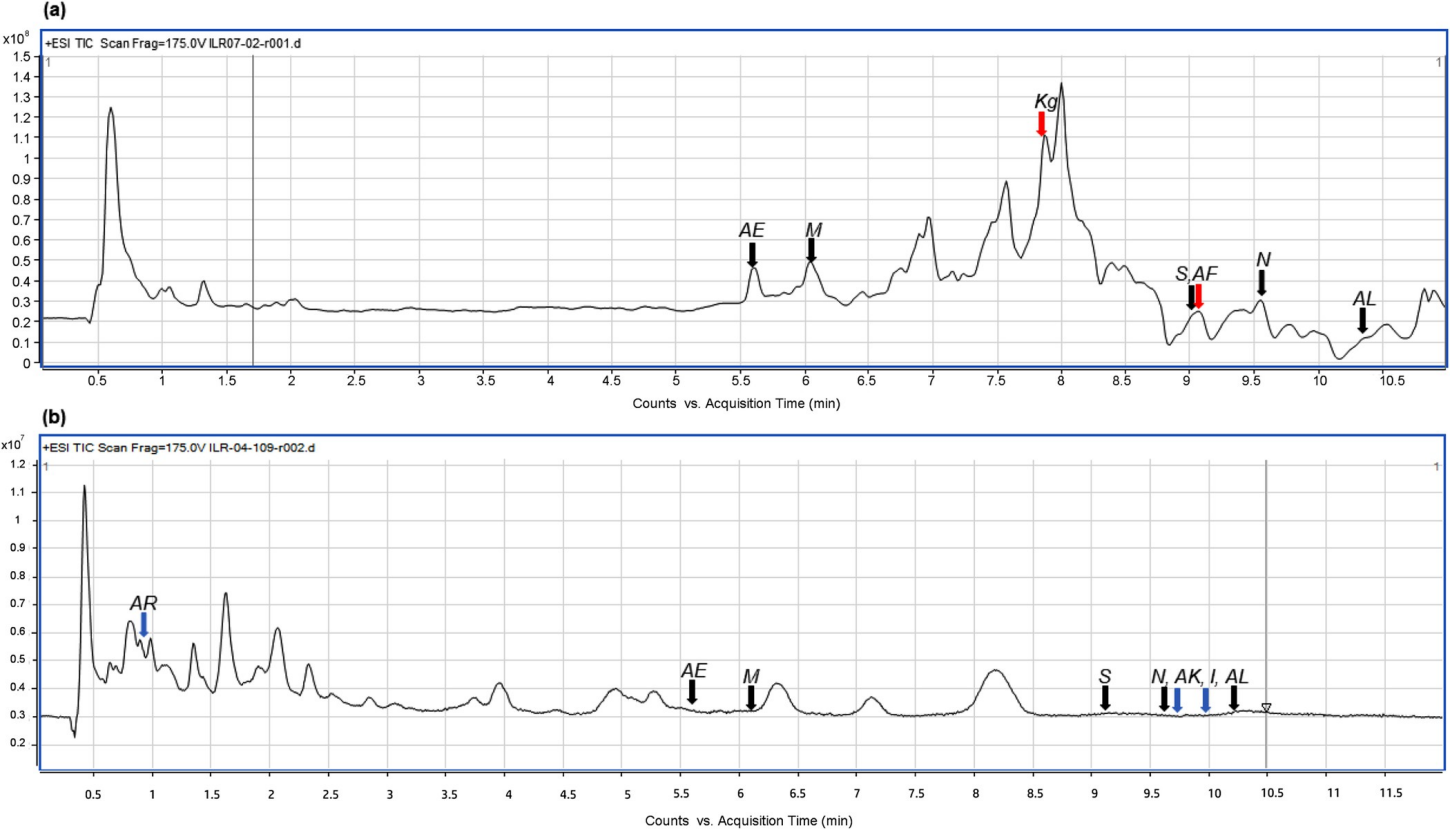
TIC chromatograms of latex of *I*. *parasitica* collected from Yautepec (A) and Tepoztlan (B). Column: Phenomenex Luna® C_18_ (50 mm × 2,6 mm d.i.; 3,0 μm). Black arrows correspond to resin glycosides found in both localities, and colored arrows correspond to resin glycosides exclusive to each site.

The content of resin glycosides of both localities was different, as it is shown by chromatograms (Fig 1A and 1B). Evidence of hydroxyacyl group bonded with sugars in all resin glycosides was obtained; C16-OH being the most common, and the unusual 11th position hydroxylation in the hydroxyacyl chain reveals the unique biochemistry of resin glycosides. Among the acyl chains, five-carbon long chains 2-methylbutyric acid and 3-hydroxy-2-methylbutyric acid were the most frequent (S1-S10 Figs in S1 File). Based on Smith et al.’s proposed structure [29], 44 resin glycosides were detected in the latex of *Ipomoea parasitica*, and those that correlated significantly with some PC were tentatively characterized (S1-S10 Figs in S1 File).

**Fig 2:**
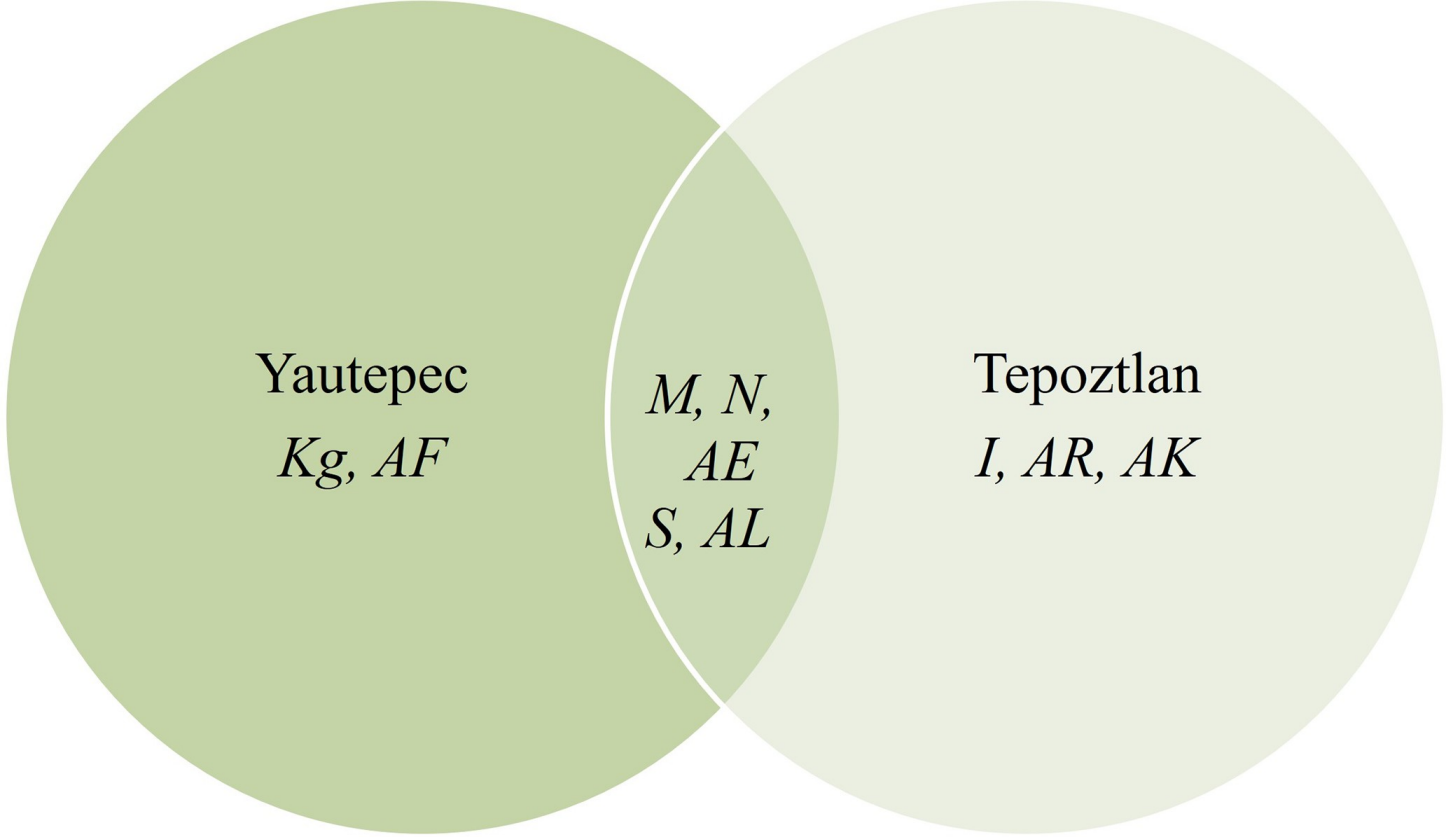
Distribution of ten resin glycosides that were significantly correlated with three PC (total variance = 47.08%). 50% were in plants from both sites, but exclusive resin glycosides were also per site (Tepoztlan: *I*, *AK*, *AR*; Yautepec: *K*^*g*^, *AF*). Venn diagram was elaborated using MS excel 365, Academic.

Ten resin glycosides correlated with three components (total explained variance = 47.07%). PC1, PC2, and PC3 explained 17.63%, 14.81%, and 14.62% respectively. Five of the ten resin glycosides were found in Tepoztlan and Yautepec (*M*, *N*, *S*, *AE* and *AL*), three were in the latex of plants located in Tepoztlan (*I*, *AK*, and *AR*), and *K*^*g*^ and *AF* were found in Yautepec (Fig 2). The tentative number of sugar moieties in these resin glycosides ranges from three to five bonded with one to three fatty acids. Three of the resin glycosides had two dimmers of tetrassacharide, two of them (*AL* and *AR*) were in latex of Tepoztlan plants (S1-S10 Figs in S1 File).

**Fig 3:**
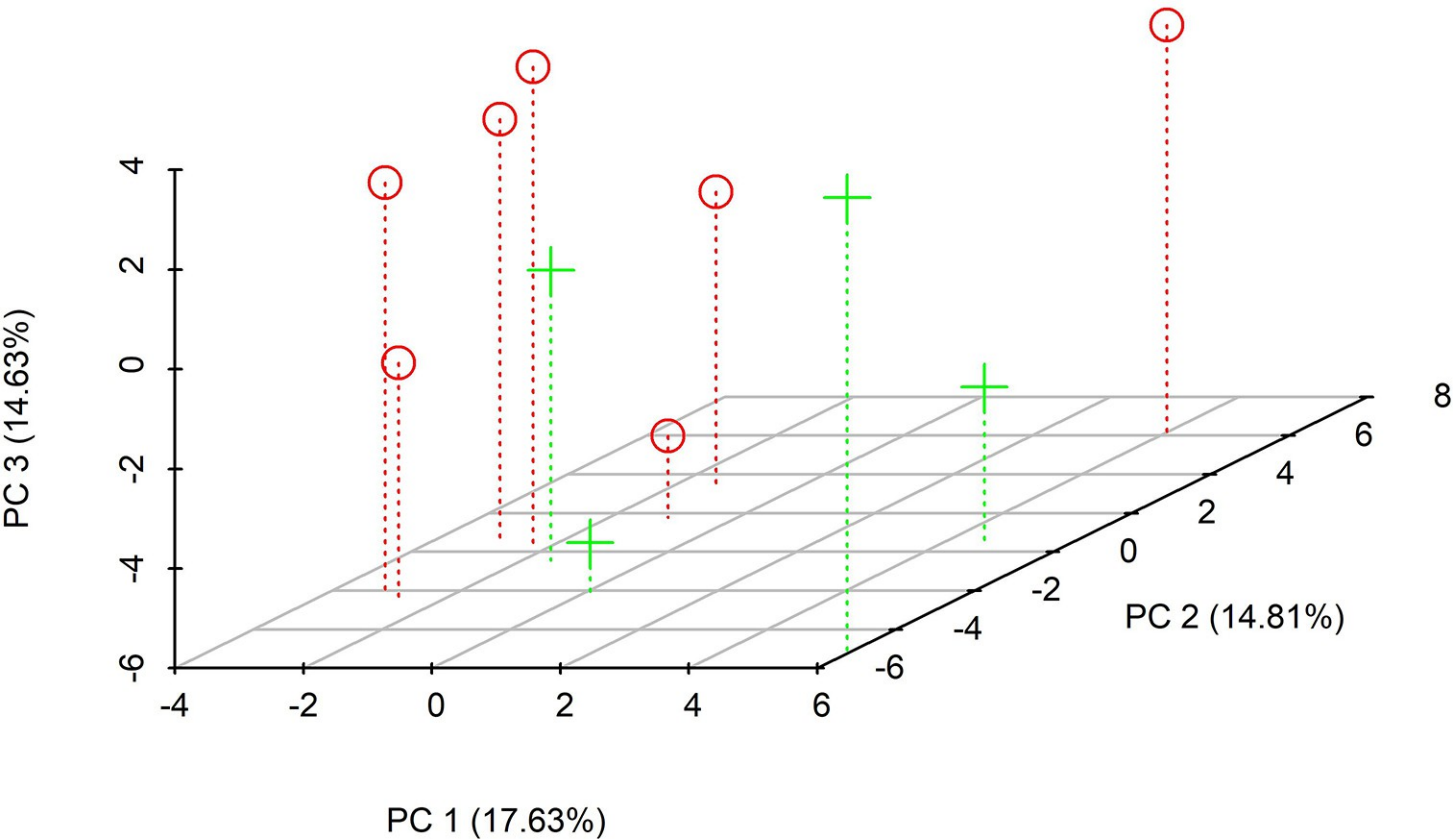
Distribution in three PCs of eleven *I*. *parasitica* plants from two sampling sites (Tepoztlan and Yautepec). The library Scatterplot3d [46] was employed to construct this graph.

**Fig 4:**
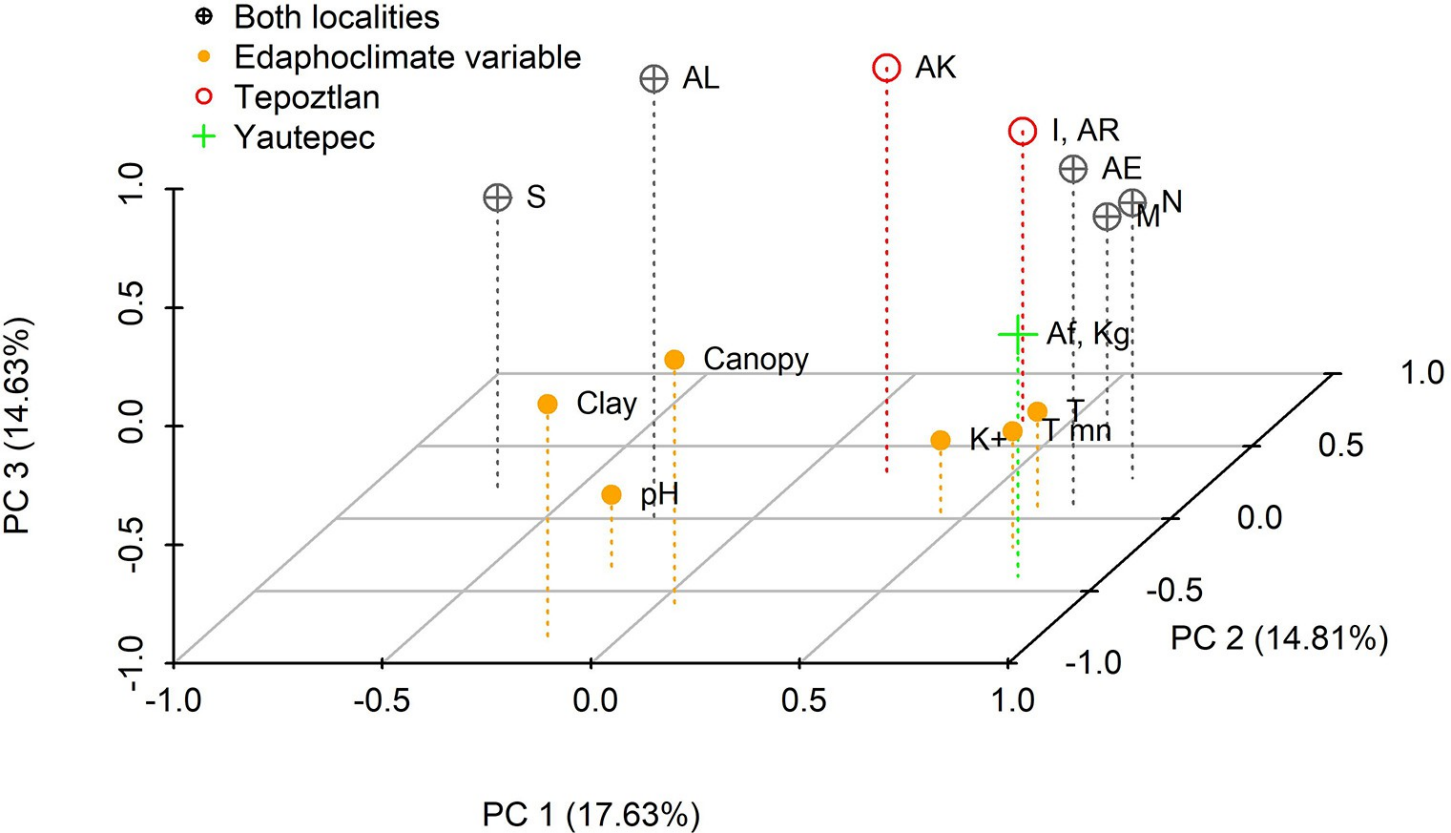
Significant correlations of 10 resin glycosides of *I*. *parasitica* latex and edaphoclimate variables with the PC. Resin glycosides *K*^*g*^, *M*, *N*, *S AE*, *AF* and minimum and average temperatures correlated with PC1. Compounds *I*, *AR*, % clay and % canopy correlated with PC2 and the PC3 correlated with *AK*, *AL*, K^+^ and pH. Different colors and symbols correspond to glycolipids that were Tepoztlan, Yautepec or both localities. Edaphoclimate variables are also indicated. The library Scatterplot3d [46] was employed to construct this graph.

The main pattern is observed in PC1, where the separation of plants mainly depends on the locality. The negative axis of this PC integrates six plants from Tepoztlan. Meanwhile two Yautepec´s plants correlated positively (Fig 3). Resin glycosides that correlated positively with this component were *K*^*g*^ (*m/z* = 1131.6026, *r = 0*.*79*), *M* (*m/z* = 1149.6129, *r = 0*.*65*), *N* (*m/z* = 1157.6893, *r = 0*.*80*), *AE* (*m/z* = 1337.6890, *r = 0*.*74*) and *AF* (*m/z* = 1347.8949, *r = 0*.*79*). The PC1 also was positively correlated with the minimum and average temperatures (*r = 0*.*70* and *r = 0*.*65*, respectively). Resin glycoside identified as *S* was negatively associated with PC1 (*m/z* = 1247.6815, *r = -0*.*69*) (Table 1, Fig 4). As can be seen, most of the resin glycosides correlated with the first component, highlighting that those from the same locality tend to be grouped with one single PC (Table 1, Figs 2 and 4).

**Table 1:** Correlations between variables and the principal components (PC). High correlations (r ≥ |0.65|) were marked in bold. The letters in the variable correspond with the compounds shown in S1 File. $\bar{T}$ = average temperature.

Variable	PC1	PC2	PC3
*I*	0.39	**0.66**	0.23
*K* ^*g*^	**0.79**	-0.40	0.02
*M*	**0.65**	0.51	-0.04
*N*	**0.80**	0.28	0.16
*S*	**-0.69**	0.20	0.23
*AE*	**0.74**	0.07	0.43
*AF*	**0.79**	-0.40	0.02
*AK*	0.20	0.31	**0.71**
*AL*	-0.24	0.006	**0.85**
*AR*	0.39	**0.66**	0.23
K^+^	0.45	0.00	**-0.67**
Clay	-0.17	**-0.83**	-0.01
Ph	-0.20	-0.36	**-0.68**
Canopy	0.06	**-0.64**	0.06
Temp. min	**0.70**	-0.20	-0.51
$\bar{T}$	**0.65**	0.08	-0.60

The resin glycosides *I* (*m /z* = 1115.6434, *r = 0*.*66*) and *AR* (*m/z* = 2277.2356, *r = 0*.*66*) were positively associated with PC2. Meanwhile, it is observed negative correlations with the percentage content of clay in the soil (*r = -0*.*83*) and the canopy (*r = -0*.*65*) (Table 1, Fig 3). K^+^ (*r = -0*.*67*) and pH (*r = -0*.*66*) correlated negatively with PC3. Resin glycosides that correlated significantly and positively with this component were *AK* (*m/z* = 1881.5570, *r = 0*.*71*) and *AL* (*m/z* = 1903.0852, *r = 0*.*85*) (Table 1, Fig 4).

### Edaphoclimate

Soil characteristics reflected similarities between both localities (Table 2). For example, the average organic matter percentage ranged between 2.21% and 2.97% for Tepoztlan and Yautepec, respectively. The BD of both study sites (1.01 ± 0.14 g/cm3) corresponds to clay soil according to the NOM-021-RECNAT 2000 [40]. In addition, *I*. *parasitica* was found in soils with a comparatively less acidic pH (6.57 to 6.78), with a rockiness of 7.08 ± 10.54%, plant cover of 86.66 ± 25.70%, and surrounded by Burseraceae and Fabaceae species. Canopy coverage was 45.68 ± 28.76%, and soil moisture 4.26 ± 0.95%. Nevertheless, soil profundity was deeper in Yautepec than in Tepoztlan (Table 2).

**Table 2:** Physical-chemical characteristics (mean ± 1SD) of the soil where *I*. *parasitica* grows. Mann-Whitney *U* values are indicated for each environmental parameter. The minimum and maximum ranges are shown in parentheses within the level column. Those references different from NOM-021-RECNAT-2000 [40] are shown (see moisture and rockiness parameters). NA = Not applicable.

Soil parameter	Tepoztlan	Yautepec	Total	Reference level
OC (%)	1.48 ± 0.46	2.018 ± 0.87	1.72 ± 0.68	Low (<11)
(*U = 6*.*00*, *P = 0*.*25*)				
N (%)	0.19 ± 0.4	0.33 ± 0.19	0.23 ± 0.13	High (0.15–0.25)
(*U = 5*.*00*, *P = 0*.*18*)				
K^+^ (ppm)	51.21 ± 29.63	55.56 ± 16.14	52.17 ± 17.44	Very low (<150)
(*U = 5*.*00*, *P = 0*.*50*)				
Ca^2+^(ppm)	1244.92 ± 1687.28	2612.46 ± 871.65	1802.58 ± 1433.63	Average (1000–2000)
(*U = 3*.*00*, *P = 0*.*18*)				
Mg^2+^(ppm)	117.54 ± 61.64	159.26 ± 40.84	134.M76 ± 52.54	Low (60–158)
(*U = 3*.*00*, *P = 0*.*18*)				
Na^+^(ppm)	9.31 ± 1.20	8.72 ± 0.97	9.06 ± 1.07	Very low (optimum < 30–60
(*U = 6*.*50*, *P = 0*.*57*)				
Moisture (%)	3.97 ± 0.63	4.75 ± 0.97	4.26 ± 0.95	Maximum ~18 (0–20 cm of soil depth [46]
(*U = 3*.*00*, *P = 0*.*34*)				
BD (g/cm^3^)	0.98 ± 0.07	1.04 ± 0.19	1.01 ± 0.14	1–1.19
(*U = 7*.*00*, *P = 0*.*70*)				
TOC (%)	1.24 ± 0.74	1.72 ± 0.56	1.47 ± 0.68	Low (<11)
(*U = 7*.*00*, *P = 0*.*25*)				
OM (%)	2.19 ± 1.27	2.97 ± 0.98	2.53 ± 1.17	Rank (1.6–3.5)
(*U = 7*.*00*, *P = 0*.*25*)				
Silt (%)	27.5 ± 19.95	29 ± 6.78	28.16 ± 13.92	NA
(*U = 6*.*00*, *P = 0*.*63*)				
Clay (%)	44.20 ± 10.85	50 ± 7.07	46.91 ± 9.39	NA
(*U = 5*.*50*, *P = 0*.*18*)				
Sand (%)	28.29 ± 20.22	21.20 ± 5.06	25 ± 14.56	NA
(*U = 7*.*50*, *P = 0*.*85*)				
pH	6.57 ± 0.65	6.78 ± 0.12	6.67 ± 0.45	Neutral (6.6–7.3)
(*U = 4*.*00*, *P = 0*.*25*)				
P (ppm)	45.28 ± 36.86	54.02 ± 36.29	48.81 ± 33.71	High (>30)
(*U = 6*.*00*, *P = 0*.*57*)				
Depth (cm)	16.12 ± 10.67	30.00 ± 0.00	21.91 ± 8.65	NA
(*U = 0*.*00*, *P < 0*.*001*)				
Rockiness (%)	4.79 ± 12.02	10.00 ± 12.24	7.08 ± 10.54	Low (82.17 ± 25) [47] and (43.3–81.1) [48])
(*U = 6*.*00*, *P = 0*.*50*)				
Plant cover (%)	82.08 ± 22.62	96.00 ± 8.94	86.66 ± 25.70	NA
(*U = 2*.*00*, *P = 0*.*13*)				
Canopy (%)	33.26 ± 13.28	57.50 ± 19.03	45.68 ± 28.76	NA
(*U = 8*.*00*, *P = 0*.*25*)				

The climatic characteristics of Tepoztlan and Yautepec were similar (Table 3); however, it is observed that the maximum temperatures in Tepoztlan were three degrees above the maximum in Yautepec.

**Table 3:** Average climatic variables of Yautepec and Tepoztlan recorded from August to November 2019. $\bar{T}$ = average temperature, PP = rainfall.

Locality	Temp. Max. (°C)	Temp. min. (°C)	$\bar{T}$ (°C)	PP (mm)
Tepoztlan	30.85	16.45	18.07	5.8
Yautepec	27.82	17.62	18.55	4.1
Mean ± SD	29.69 ± 1.84	16.94 ± 0.79	18.23 ± 0.55	5.05 ± 2.92

## Discussion

### Phytochemical analysis of latex

As in other resin glycosides across *Ipomoea* species [49], the glycolipids found here showed variation among the number of monosaccharide units (tetra, penta, and dimers of tetrasaccharide) besides the acyl groups bonded to the saccharide core. Longer saturated acyl chains were not detected, indicating the predominant role of branched-chain amino acid biosynthesis, from which most of the small acyl chains are likely derived in resin glycosides. As in Solanaceae, this structural variation may be due to the BAHD acyltransferases’ promiscuity in their biosynthesis [5, 50]. In other types of acylsugar, BAHDs show conservation of acylation positions in orthologs despite their acceptor promiscuity; however, in resin glycosides, analysis of previously characterized structures does not suggest positional uniformity [51].

The structures of resin glycosides found here are based on the data reported in the scientific literature [51], like macrolactones, glycosidic acids, or dimers, which usually contain the same carbon skeleton or substructures, thus the same fragment ions could be defined as diagnostic fragment ions in mass spectrometry. Analysis of the pseudo-molecular ion masses of resin glycosides isolated and characterized by our research group revealed that *Ipomoea* species produced intermediate and high molecular weight (MW) monomer glycolipids (800 <MW<1600). This finding agrees with previously reported resin glycosides, containing predominantly tri-, tetra-, and pentasaccharides from *Ipomoea* species [49].

The resin glycosides reported for the genus *Ipomoea* have high structural complexity. Obtaining pure compounds is a challenge that requires advanced chromatographic techniques, such as core cutting and sample recycling in HPLC [52]. The present work aims to identify glycolipids in *I*. *parasitica* through UHPLC-MS. The fragmentation patterns indicate the presence of monohydroxy and dihydroxy C14 and C16 fatty acids, as well as structures with different chain lengths (tiglic, isobutyric, methylbutyric, decanoic and dodecanoic acids), epimers of hexoses (glucose, mannose, and galactose) and pentoses (rhamnose, fucose, and quinovose) that are ester-linked to the oligosaccharide cores, forming macrolactone ring spanning two or more units (S1-S10 Figs in S1 File). These chemical features may allow us to conclude the presence of glycolipids [51]. However, like Smith and Cols. [29], the amount of latex obtained from *I*. *parasitica* was insufficient to characterize these molecules. Hence, before future phytochemical studies, strategies must be implemented that make latex production and collection more efficient (i.e., tissue culture).

### Edaphoclimate

Environmental stressors elicit in plants the elaboration of molecules that participate in plant defense, like those contained in latex [53], as was documented in *Hevea* species, where environmental conditions influence the expression of genes involved in glycolipid biosynthesis [54]. Although the resin glycosides observed in the latex of *I*. *parasitica* could be synthesized in response to certain edaphic and climatic conditions, apparently there are not a clear relation between a specific chemical structure with a particular edaphic or climatic condition, even considering that two of the three resin glycosides with tetrasaccharide dimers were in Tepoztlan.

The PC ordination analysis showed that ten of the 44 resin glycosides found in the latex of *I*. *parasitica* correlated (r ≥ |0.65|) with one of the three components. Of these, 50% were present in both sampling locations, indicating the possibility of being constitutive metabolites, which are usually found in a preformed form in plants, regardless of environmental factors [55]. On the contrary, exclusive resin glycosides were obtained from plants of each locality, which could explain genetic differences between populations spatially separated. It has been reported that latex from accessions of *Solanum penelli* Correll, have differences in their acylsugar proportion, attributing this difference to a mixed distribution of functional and nonfunctional alleles IPMS3 [5]. Differences in the yield and composition of resin glycosides from *I*. *tyrianthina* plants among different geographical areas were also observed [8]. However, this could be attributable also to differences in biotic and abiotic factors.

An environmental elicitor could also induce the exclusive resin glycosides found in the present study. Glycolipids were correlated with some components, and edaphoclimate variables correspond to tetrasaccharide resin glycosides bonded to jalapinolic acid. The PCA suggests that rising temperatures (minimum and average) were positively correlated with the glycolipids *K*^*g*^, *AF*, and *M*, *N*, *AE*;. At the same time, the resin glycoside *S* was present in lower temperatures. Different studies have shown that the presence of some glycolipids is related to extreme temperatures [56, 57], possibly because of their protective role in maintaining membrane fluidity. Plants stabilize their glycolipid content through “homeoviscose adaptation”, which refers to the maintenance of the fluid consistency of the membranes in extreme temperatures [58]. Low temperatures cause an increase in glycolipid precursors and glycolipids of diverse families of monogalactosyldiacyglycerol (MDGD), digalactosyldiacyglycerol (DGDG) and sulfoquinovosyldiacylglycerol (SQDG) [57] that contain polyunsaturated fatty acids that maintain membrane fluidity [58, 59]. On the contrary, if the temperature increases, the phospholipids increase over the glycolipids, whose molecules would have saturated fatty acids [58]. Glycolipids such as monogalactosyldiacyglycerol and digalactosyldiacyglycerol are not only structural in the membrane system of the photosynthetic apparatus but are also found in the latex of species such as *Hevea brasiliensis*, where digalactosyldiacyglycerol is the majority component of the latex (43–51%) [54].

Resin glycosides *I* and *AR* were found in the latex collected in Tepoztlan. According to PCA, these molecules were present in soils with a lower percentage of clay and sites with lower canopy. The canopy creates microclimatic conditions below it, which facilitates the presence of other organisms [60] by lowering the average temperature at least 2°C compared to open sites with greater light intensity [61]. Therefore, since there is a low canopy in the study site, it is possible that the incidence of light and high temperature could act as elicitors of these compounds (*I* and *AR*). Indeed, it is demonstrated that N is released under canopy when leaves fall, suggesting that the dynamic of N cycle could depend on the blue light that passes through the canopy [62]. Other factors, such as N mineralization, stimulate the production of fatty acids, and resin glycosides have fatty acids [63]. N mineralization is higher in canopy gaps [64, 65]; it is likely that the canopy found here (<50%) was sufficient to create “fertility islands” [66] and would favor the presence of compounds *I* and *AR*, which were also present in less clayey soils.

The soil is a porous matrix that covers the earth, made up of mineral particles and organic matter, where organisms interact, modifying its physicochemical characteristics; for example, plant roots break down minerals in the soil, increasing its porosity and moisture [17, 67]. Soil may have characteristics that do not favor plant growth, such as hardness, large pores, and high bulk density [17]. Clay soils have a particle size <2 μm, which facilitates high bulk density and low porosity; however, it has good water retention [68]. If less clayey soils retain less moisture, then stressful conditions would be created that, together with an open canopy (high temperature), would favor the presence of compounds *I* and *AR*, which could be involved in the defense against these factors.

Compound *AK* showed a positive correlation with pH (from neutral to slightly acidic) and a negative correlation with K^+^. In a comparative study of three natural amendments (K-humate, dry-vermicast and volcanic minerals), it was found that amendment concentrations with less K^+^ concentration (0.2 mg/g, K-humate) and acidic pH were related to increased sulfoquinovosyldiacylglycerol with monounsaturated fatty acids (C16:1 and C18:1) and polyunsaturated C18:2 and C18:3 in *Brassica oleracea* var. *acephala* D.C. [67]. Something similar occurred in *I*. *parasitica*: K^+^ deficiency and slightly acidic soil pH (6.67 ± 0.45) were related to compounds *AK* and *AL*. Both studies suggest that glycolipid variation in plants may reflect the nutrient composition of the growing medium and climatic conditions.

## Conclusions

Here, we reported for the first time the resin glycoside profile of this species (tetrasaccharide, pentasaccharide, and dimers of tetrasaccharide units) and the climatic and edaphic factors associated with these molecules. There were detected 44 resin glycosides in the latex of *I*. *parasitica* plants located in two sites of central Mexico. Temperature, % clay, pH, K^+,^ and 10 resin glycosides contributed to explaining the grouping patterns of plants, which were separated mainly for the locality; three resin glycosides were exclusive to Tepoztlan, and two to Yautepec, meanwhile five molecules were found in both localities and considered constitutive. This research provides the basis for the chemical structure of resin glycosides, but complementary studies are needed to propose names. Moreover, the results found here will serve as a platform to delve into the ecology, biochemical, and molecular basis of their biosynthesis and to develop biotechnological strategies to obtain these molecules considering specific substrate characteristics.

## Supporting information

S1 File Supplementary material (S1 File) includes S1-S10 Figs that correspond to the chemical characterization of the ten resin glycosides that correlated with the three PC and with a range of molecular masses from 1115 to 2277. (DOCX)
