# Supplementary material for: Effect of edaphoclimate on the resin glycoside profile of the ruderal Ipomoea parasitica (Convolvulaceae)
Source: PLoS One. 2024 Aug 8;19(8):e0305003. doi: 10.1371/journal.pone.0305003 (PMC11309378; doi:10.1371/journal.pone.0305003)
Supplement: S1 File — (DOCX) [file pone.0305003.s001.docx]

**Supporting information**

**S1 Appendix**. Chemical characterization of the ten resin glycosides that correlated with the three PC and with a range of molecular masses from 1115 to 2277.

**S1 Fig Resin glycoside *I* (Tepoztlan).** Chemical characterization (UHPLC- ESI-Q-TOF). In the high-resolution positive FAB modality, presented a quasi-molecular ion [M+H^+^]^+^ at *m/z =* 1299.7127. The signal at *m/z* = 1217.6700 and 1115.6434 corresponded to the consecutive loss of one unit of methylbutanoyl (C_5_H_6_O) and one unit of 2-methylbutanoic (C_5_H_10_O_2_), *m/z* = 857.6714 [1115 – 258 (C_16_H_34_O_2_)], *m/z* = 799.5364 [857 – 58 (C_3_H_6_O)], *m/z* = 589.4276, *m/z* = 447.3496 and *m/z* = 402.2505.

**S2 Fig** **Resin glycoside *K^g^* (Yautepec).** Chemical characterization (UHPLC- ESI-Q-TOF), presented a quasi-molecular ion [M+H^+^]^+^ at *m/z* = 1268.7819. The signal at *m/z* = 1131.6026 corresponded to the loss of two C_4_H_5_O units, *m/z* = 982.6027 [1131 – 149 (methylpentose unit) (C_6_H_13_O_4_)], *m/z* = 633.3313, *m/z* = 443.3382 and *m/z* = 316.9325.

**S3 Fig** **Resin glycoside *M* (Tepoztlan and Yautepec).** Chemical characterization (UHPLC- ESI-Q-TOF) presented a quasi-molecular ion [M+H^+^]^+^ at *m/z* = 1211.5838. The mass spectrum showed signal at *m/z* = 1149.6129, *m/z* = 815.6073, *m/z* = 719.4417 [815 – 96 (hexanoyl) (C_6_H_8_O)], *m/ z =* 535.3655, *m/z* = 402.2531 [535 – 133 (cinnamoyl) (C_9_H_9_O)], *m/z* = 297.2431 [402 – 105 (2-methylbutanoic) (C_5_H_13_O_2_)] and *m/z* = 214.9198 [297 – 83 (methylbutanoyl) (C_5_H_7_O)].

**S4 Fig** **Resin glycoside *N* (Tepoztlan and Yautepec).** Chemical characterization (UHPLC- ESI-Q-TOF) exhibited the quasi-molecular ion [M+H^+^]^+^ at *m/z* = 1287.7532. The signal at *m/z* = 1157.6893 corresponded to the loss of one unit of cinnamoyl (C_9_H_6_O), *m/z* = 793.4417 [1157 – 364 (2 x C_12_H_22_O)], *m/z* = 689.5353 [793 – 104 (3-hydroxy-2-methylbutanoyl) (C_5_H_12_O_2_)], *m/z* = 402.2526 [689 – 287 (2 x 2-methylbutanoic, C_5_H_10_O_2_) + (methylbutanoyl) (C_5_H_9_O)] and *m/z* = 338.3444.

**S5 Fig** **Resin glycoside *S* (Tepoztlan and Yautepec)**. Chemical characterization (UHPLC- ESI-Q-TOF) exhibited the quasi-molecular ion [M+H^+^]^+^ at *m/z* = 1566.0864. The signal at *m/z* = 1257.6815 corresponded to the loss of one unit of C_6_H_7_O_3_ and one unit of C_12_H_22_O, *m/z* = 1025.7361 [1247 – 222 (C_4_H_4_O + decanoyl, C_10_H_18_O)], *m/z* = 793.4379 [1025 – 232 (methylpentose unit) (C_6_H_11_O_4_) + (methylbutanoyl) (C_5_H_9_O)], *m/z* = 705.3860 [793 – 88 (C_5_H_12_O)], *m/z* = 603.4467 [705 – 102 (2-methylbutanoic, C_5_H_10_O_2_)], *m/z* = 515.3938 [603 – 88 (C_5_H_12_O)], *m/z* = 417.3678, *m/z* = 402.2504 and *m/z* = 326.1043.

**S6 Fig** **Resin glycoside *AE* (Tepoztlan and Yautepec).** Chemical characterization (UHPLC- ESI-Q-TOF) exhibited the quasi-molecular ion [M+H^+^]^+^ at *m/z* = 1337.6890. The mass spectrum showed a signal at *m/z* = 1275.7199, *m/z* = 646.3463, *m/z* = 570.9345, *m/z* = 402.2530 [570 – 168 (2-methylbutanoic, C_5_H_8_O_2_) + (C_4_H_4_O)]. Cleavage of glycoside bond produced diagnostic ion at *m/z* = 270.3185.

**S7 Fig** **Resin glycoside *AF* (Yautepec).** Chemical characterization (UHPLC- ESI-Q-TOF) exhibited the quasi-molecular ion [M+H^+^]^+^ at *m/z* = 1347.8949. The signal at *m/z* = 1233.6693 corresponds to the loss of two C_3_H_5_O units, *m/z* = 955.4288 [1233 – 278 (methylpentose unit, C_6_H_10_O_4_) + (cinnamoyl) (C_9_H_8_O)], *m/z* = 868.6064 [955 – 87 (methylbutanoyl) (C_5_H_11_O)], *m/z* = 663.4599 [762 – 99 (3-hydroxy-2-methylbutanoyl, C_5_H_7_O_2_)], *m/z* = 570.9349 y *m/z* = 402.2529.

**S8 Fig** **Resin glycoside *AK* (Tepoztlan).** Chemical characterization (UHPLC- ESI-Q-TOF) exhibited the quasi-molecular ion [M+H^+^]^+^ at *m/z* = 1881.5570. The signal at *m/z* = 1624.1330 corresponded to the loss of one unit of C_16_H_33_O_2_, *m/z* = 1338.8137 [1624 – 286 (2 x 2-methylbutanoic, C_5_H_10_O_2_) + C_5_H_6_O], *m/z* = 1261.6983, *m/z* = 1084.7813, *m/z* = 845.6015 [1084 – 239 (C_3_H_5_O + C_12_H_22_O)], *m/z* = 522.5993 and *m/z* = 402.2513.

**S9 Fig** **Resin glycoside *AL* (Tepoztlan and Yautepec).** Chemical characterization (UHPLC- ESI-Q-TOF) exhibited the quasi-molecular ion [M+H^+^]^+^ at *m/z* = 1903.0852. The mass spectrum showed signal at *m/z* = 1491.0087, *m/z* = 1352.8402, *m/z* = 1275.7177, *m/z* = 1190.7859 [1275 – 85 (methylbutanoyl, C_5_H_9_O)], *m/z* = 1108.7083 [1190 – 82 (methylbutanoyl, C_5_H_6_O)], *m/z* = 728.6084, *m/z* = 522.6084, *m/z* = 453.3386 [522 – 69 (C_4_H_5_O)] and *m/z* = 402.2532.

**S10 Fig** **Resin glycoside *AR* (Tepoztlan).** Chemical characterization (UHPLC- ESI-Q-TOF) exhibited the quasi-molecular ion [M+H+]+ at *m/z* = 2277.2356. The mass spectrum shows signal at *m/z* = 1149.6147, *m/z* = 1021.5635 [1149 – 128 (C_3_H_4_O + C_4_H_8_O)], *m/z* = 817.5011 [1021 – 204 (2 x 2-methylbutenoic, C_5_H_10_O_2_)], *m/z* = 671.4186 [817 – 146 (methylpentose unit, C_6_H_10_O_4_)], *m/z* = 583.2936, *m/z* = 519.2964, *m /z* = 365.2804 [519 – 154 (decanoyl, C_10_H_18_O)] and *m/z* = 214.9197.
